# Abstracts

**Published:** 1882-12-20

**Authors:** 


### "*ABSTRACTS.*"

Among the changes that are to be found in the new United States Pharmacopœia is a class of preparations to which has been given the name of *Abstracts*. They are in the form of a powder, prepared by evaporating a concentrated tincture of the drug, having first mixed it with sugar of milk.

The solid extract of most drugs, as now used, often proves unreliable by reason of deterioration. These Abstracts are apt to take the place of solid extracts, as being of more uniform strength and more easily preserved.

